# Flow-Through Acetylcholinesterase Sensor with Replaceable Enzyme Reactor

**DOI:** 10.3390/bios12090676

**Published:** 2022-08-24

**Authors:** Alexey Ivanov, Dmitry Stoikov, Insiya Shafigullina, Dmitry Shurpik, Ivan Stoikov, Gennady Evtugyn

**Affiliations:** 1 A.M. Butlerov’ Chemistry Institute, Kazan Federal University, 18 Kremlevskaya Street, 420008 Kazan, Russia; 2 Analytical Chemistry Department, Chemical Technology Institute, Ural Federal University, 19 Mira Street, 620002 Ekaterinburg, Russia

**Keywords:** acetylcholinesterase sensor, inhibitor determination, flow-through analysis, electropolymerization, pillar[5]arene

## Abstract

Fast and reliable determination of enzyme inhibitors are of great importance in environmental monitoring and biomedicine because of the high biological activity and toxicity of such species and the necessity of their reliable assessment in many media. In this work, a flow-through biosensor has been developed and produced by 3D printing from poly(lactic acid). Acetylcholinesterase from an electric eel was immobilized on the inner walls of the reactor cell. The concentration of thiocholine formed in enzymatic hydrolysis of the substrate was monitored amperometrically with a screen-printed carbon electrode modified with carbon black particles, pillar[5]arene, electropolymerized Methylene blue and thionine. In the presence of thiocholine, the cathodic current at −0.25 V decreased because of an alternative chemical reaction of the macrocycle. The conditions of enzyme immobilization and signal measurements were optimized and the performance of the biosensor was assessed in the determination of reversible (donepezil, berberine) and irreversible (carbofuran) inhibitors. In the optimal conditions, the flow-through biosensor made it possible to determine 1.0 nM–1.0 μM donepezil, 1.0 μM–1.0 mM berberine and 10 nM to 0.1 μM carbofuran. The AChE biosensor was tested on spiked samples of artificial urine for drugs and peanuts for carbofuran. Possible interference of the sample components was eliminated by dilution of the samples with phosphate buffer. Easy mounting, low cost of replaceable parts of the cell and satisfactory analytical and metrological characteristics made the biosensor a promising future application as a point-of-care or point-of-demand device outside of a chemical laboratory.

## 1. Introduction

Growing contamination of the environment with pesticides and drug residues and strict limitations on potential hazardous content in drinking water and foodstuffs call for the development of simple and reliable analytical devices intended for preliminary screening of potential pollutants and for the assessment of their exposure to humans outside chemical laboratories [1,2]. They are considered as an alternative to conventional sophisticated analytical instrumentation such as HPLC or fluorescence spectroscopy to provide information on the chemical content of soil, water, agriculture and food industry samples that is requested for making decisions on safety and potential risks related to xenobiotics and the products of their conversion in the environment. Such portable analytical devices have some specific requirements related to the application area. Thus, they should be portable, rather compact and assume minimal sample treatment and consumables consumption [1]. Group assessment of the analytes in accordance with their similar chemical structure or adverse effect is also desirable [2].

Among various approaches to the preliminary testing of chemical hazards, biosensors offer unique opportunities for risk assessment [3]. In these analytical devices, biological elements (enzymes, nucleic acids, antibodies) are implemented in the assembly of a specific transducer that converts a biochemical event into the electric signal measured and interpreted in terms of the analyte content or its activity in an appropriate reaction [4]. Due to the extraordinary selectivity of most biochemical recognition reactions, biosensors can be used in rather complex media including biological fluids in on-site and online regimes. To some extent, biochemical reactions responsible for target recognition in the assembly of biosensors mimic appropriate processes that take place in organisms and can be considered the simplest model of toxic effects caused by contaminants [5]. Similar methodology can be extended to the determination of drug residuals that also exert targeted effects related to certain biochemical paths and are under strict control, especially for rather toxic species with a narrow gap between the toxic and pharmaceutical dose [6,7]. Thus, biosensors offer a variety of advantages over conventional chemical analysis that can be rather simply realized in a format convenient for on-demand use.

Although many methods have been explored for sensitive and selective detection of biochemical interactions in the biosensor assembly, electrochemical transducers remain on top of real application examples [8]. Starting from the first success related to glucometers, amperometric and impedimetric sensors are most investigated and used in biosensor prototypes. They offer many well-known advantages over optic and physical sensors, i.e., rather simple design, sufficient sensitivity of the signal measurement, well-elaborated theory, intuitively understandable interpretation of the result, low cost of primary equipment compatible with flow-through and portable modes [9,10]. Electrochemical biosensors can be easily presented in a miniature format and are frequently used in point-of-care testing mode [11].

Acetylcholinesterase (AChE) is one of the most frequently used enzymes in biosensor assembly. Starting from chemical warfare detection [12,13], AChE biosensors have found a large application in the determination of organophosphate and carbamate pesticides inhibiting enzymes [14,15,16]. Due to a wide variety of species exerting the anticholinesterase effect, AChE biosensors were proposed as indicators of general pollution of the environment [17] and for detection of heavy metals [18], surfactants [19], fluorides [20] and organic solvents [21,22] exerting a non-specific inhibitory effect. Recently, AChE biosensors were also successfully utilized for the determination of reversible inhibitors, e.g., anti-dementia drugs [23,24] and aflatoxins [25,26].

**Figure 1:**
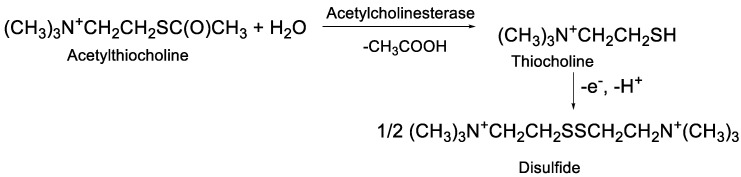
Application of ATCh for the amperometric determination of AChE activity.

Various approaches have been elaborated for the detection of AChE activity in appropriate biosensors for inhibitor determination; those most frequently used are described in reviews [14,27,28,29]. Among them, the oxidation of thiocholine formed in the enzymatic hydrolysis of acetylthiocholine (ATCh) is the most popular. The reaction (Figure 1) results in the formation of disulfide and can be monitored either amperometrically or using conventional cyclic voltammetry.

However, the reaction of thiocholine oxidation on carbon and metal electrodes is complicated by a high overvoltage and partial passivation of the electrode. For these reasons, auxiliary mediators of electron transfer are introduced in the surface layer of biosensors. Cobalt phthalocyanine [30], Prussian blue [31] Au nanoparticles [31,32], thionine [33], ferrocene [34] and Ru(II) complexes [35] were successfully utilized for this purpose. Recently, we have shown that perhydroxylated pillararenes exert electrocatalytic properties efficient for thiocholine oxidation [23,36,37]. Their effect on electron transfer is related to the reversible redox conversion of hydroquinone units of the macrocycle. However, adsorption of pillararenes on bare glassy carbon results in its fast inactivation caused by chemisorption of intermediate oxidation products. For this reason, pillararenes were implemented in the biosensor assembly by using appropriate supports such as reduced graphene oxide, carbon black or multiwalled carbon nanotubes. Although such modifiers extend the performance of appropriate biosensors, the weakened mechanical durability of the surface layer is less applicable for flow-through measurements. Implementation of electropolymerized redox active polymers as binders and mediators of electron transfer can solve this problem. Phenothiazine and diazine dyes are rather easily polymerized and entrap mediators of electron transfer on the stage of growth of the polymer film [38,39,40].

Although there are many AChE biosensors intended for the detection of inhibitors in real samples, some drawbacks limit their further application. They involve decay of the enzyme activity after each contact with an inhibitor and a multi-step protocol of biosensor assembly that involves various and incompatible conditions for the modification of primary transducer and enzyme immobilization. The use of a flow-through format and spatial separation of the enzyme layer and electrode in a thin-layer cell can solve these problems. Recently, we have proposed a simple and reliable protocol for the determination of the substrates of uricase and tyrosinase [41]. A replaceable reactor was covered with the immobilized enzyme and used as a part of the flow-through cell with a mounted screen-printed electrode modified with electropolymerized phenothiazines and pillar[5]arene (P[5]A). All the parts of the cell were produced by 3D printing from poly(lactic acid). A flow-through cell made it possible to achieve a reliable and sensitive response to uric acid and tyrosine. The assembly made it possible to quickly change both the reactor and electrode if necessary. In this work, we have extended the approach to the determination of AChE inhibitors different in nature and mechanism of interaction with the enzyme.

## 2. Materials and Methods

### 2.1. Reagents

Donepezil hydrochloride monohydrate (≥98%), berberine chloride dihydrate (≥98%), AChE from electric eel (EC 3.1.1.7, 518 U mg^−1^), ATCh, poly(lactic acid), thionine acetate (3,7-diamino-5-phenothiazinium acetate), Methylene blue (MB, 3,7-bis(dimethylamino)phenazathionium chloride), *N*-(3-dimethylaminopropyl)-*N*′-ethylcarbodiimide chloride (EDC), *N*-hydroxysuccinimide (NHS), pralidoxime (2-pyridine aldoxime methiodide, 2-PAM), and carbofuran (2,3-dihydro-2,2-dimethylbenzofuran-7-yl methylcarbamate) were purchased from Sigma-Aldrich (St. Louis, MO, USA). Unsubstituted P[5]A was synthesized at the Organic Chemistry Department of Kazan Federal University by the modified Ogoshi method [42]. Carbon black N 220 (CB, >99.95% C) was purchased from Imerys Graphite&Carbon (Willebroek, Belgium). All the working solutions were prepared using Millipore Q^®^ water (Simplicity^®^ water purification system, Merck-Millipore, Molsheim, France). Other reagents were of analytical grade.

Artificial urine contained 10 mM CaCl_2_, 6 mM MgCl_2_, 6 mM Na_2_SO_4_, 2 mM potassium citrate, 20 mM KH_2_PO_4_, 21 mM KCl, 18 mM NH_4_Cl, 9 mM creatinine and 416 mM urea.

Electrochemical measurements were performed in a 0.1 M phosphate buffer containing 0.1 M KCl.

### 2.2. Electrode Modification and Flow-Through Cell Mounting

**Figure 2:**
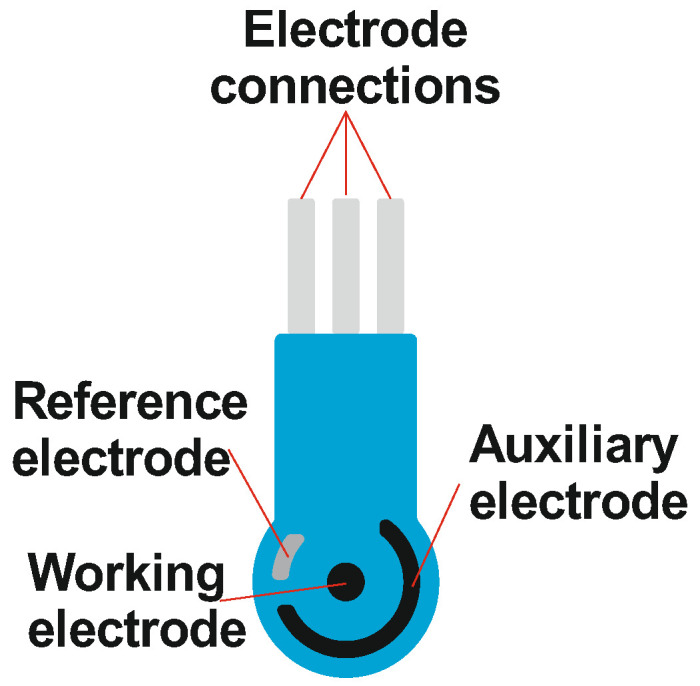
Schematic outline of screen-printed electrodes used with the flow-through cell.

Screen-printed electrodes were produced on a DEC 248 printer (DEK, London, UK) on Lomond PE DS Laser Film (thickness 125 μm, Lomond Trading Ltd., Douglas, Isle of Man). Each electrode strip included a working and an auxiliary electrode made of carbon and a reference electrode made of silver. Conducting tracks were printed using PSP-2 silver-containing paste (Delta-Paste, Moscow, Russia), carbon tracks with carbon/graphite paste C2030519P4 (Gwent group, Pontypool, UK) and the isolating layer was made of solvent-resistant blue dielectric paste D2140114D5 (Gwent group). Each layer was hardened at 80 °C. The electrode strip had dimensions of 11 × 27 mm with a geometric area of the working electrode of 3.8 mm^2^. The schematic outline of an electrode strip is presented in Figure 2. Prior to use, the working electrode was consecutively modified with the CB suspension containing P[5]A and an electropolymerized layer of the MB and thionine. The modification protocol is described in Electronic Supplementary Information (ESI).

**Figure 3:**
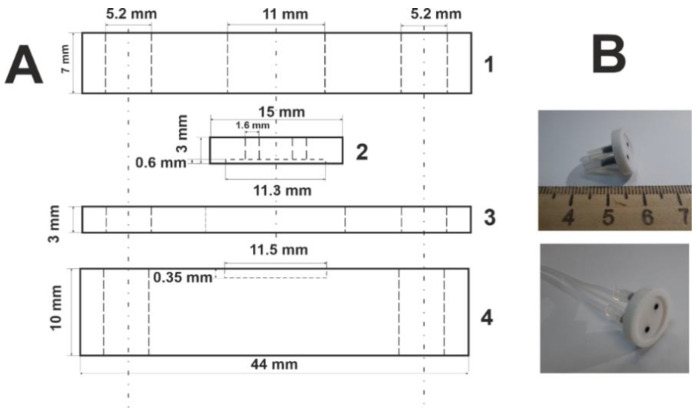
(**A**) Flow-through cell design. 1—holding lid, 2—flow-through reactor, 3—intermediate gasket, 4—base of the cell; (**B**) photographs of the reactor with plastic tubes.

The flow-through cell was prepared using Wanhao Duplicator 9/300 (Jinhua Wanhao Spare Parts Co., Jinhua, Zhejiang, China) with a single extruder (nozzle diameter 0.3 mm) from the poly(lactic acid) filaments. The 3D model of the cell designed for the printing is presented in Figure S1. Layer thickness was 0.1 mm and the printing rate was 70 mm·s^−1^. The printing temperature was 220 °C. The flow-through cell consisted of three parts fixed with two screws. The base of the cell had a rectangular notch for fixation of the screen-printed electrode strip. The replaceable enzymatic reactor contained two channels equipped with plastic tubes to pump the solutions through the cell. The schematic outline of the cell is presented in Figure 3. Photographs of a disassembled and assembled flow-through cell with a screen-printed electrode strip is presented in Figure S2.

### 2.3. AChE Immobilization and Signal Measurement

All the manipulations with the flow-through cell and signal measurements were performed at ambient temperature. The immobilization of the AChE was performed by carbodiimide binding to the inner side of a replaceable flow-through reactor (Figure 3B) by carbodiimide binding. For this purpose, 15 μL of 100 mM EDC and 15 μL of 400 mM NHS were drop-casted on the surface and left for 10 min. After rinsing with deionized water, 10 μL of the AChE solution containing 2–15 U of the enzyme were placed on the same surface, dried and rinsed again. The immobilization assumes covalent binding of the AChE molecules to the terminal carboxylic groups of the poly(lactic acid).

After assembling the flow-through cell, appropriate solutions (phosphate buffer, ATCh or an inhibitor solution) were pumped through the cell by the Model 100 Syringe Pump (ALS Co., Tokyo, Japan). The amperometric response was measured with the multi-mode potentiostat BioStat (ESA Bioscience Inc., Chelmsford, MA, USA). Electrochemical measurements in the batch conditions were performed with the electrochemical analyzer CHI 440B (CH Instruments Inc., Austin, TX, USA).

Inhibition degree was quantified as a relative decay of the signal recorded prior to and after the contact of the immobilized AChE with the inhibitor. For irreversible inhibitor (carbofuran), the solutions of the substrate and inhibitor were switched so that the enzyme contacted with the inhibitor with no substrate in the solution. Reversible inhibitors (donepezil, berberine) were added in various quantities to the 0.1 mM ATCh and influenced the immobilized AChE simultaneously. The reactivation of inhibited AChE was performed by treatment with reactivator in the case of irreversible inhibitor and washing out with phosphate buffer for reversible inhibitor.

### 2.4. Real Sample Assay

Determination of the drugs in spiked artificial was performed as described above for standard solutions of appropriate species after dilution of the samples with phosphate buffer. The dilution degree was established in blank experiments by changes in the signal related to the AChE activity.

Determination of carbofuran in peanuts was performed as follows: First, 1 g of peanuts were ground and mixed with 1 mL of acetonitrile containing a certain amount of the pesticide. After 24 h incubation, the solvent was evaporated. Prior to measurements, the dried ground sample was wetted with 1 mL of acetonitrile and after 10 min, 100 mL of phosphate buffer. Then, the liquid phase was separated and pumped through the cell as described previously for standard carbofuran solutions. In accordance with the appropriate calibration curves, extraction of carbofuran was equal to 91%.

## 3. Results

### 3.1. Modification of the Screen-Printed Electrode with Polymeric Phnothiazine Dyes and P[5]A

The conditions for the electropolymerization of the MB and thionine on the electrode have been optimized for similar flow-through conditions in our earlier work [41]. Briefly, the concentrations and polymerization conditions have been specified to reach the maximal efficiency of polymerization and stability of the redox characteristics of the coating. It was found that the use of the mixture of MB and thionine resulted in higher currents recorded against layer-by-layer deposition of each dye or the use of a monolayered film of only MB or thionine.

**Figure 4:**
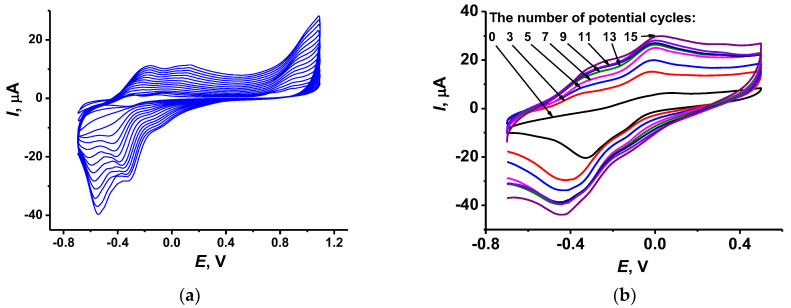
(**a**) An example of cyclic voltammograms recorded on screen-printed electrodes covered with CB and adsorbed P[5]A in the mixture of 0.1 mM MB and 0.1 mM thionine during their electropolymerization; (**b**) one cycle voltammograms recorded in working buffer with no dye monomers on the screen-printed electrodes modified with CB + P[5]A and polymerized MB and thionine, differing in the number of electropolymerization cycles performed. Measurements in 0.01 phosphate buffer + 0.1 M KCl, pH = 7.0, 100 mV/s.

The electropolymerization was performed by repeating the cycling of the potential. It resulted in the consecutive growth of the peaks attributed to the redox reactions of the dyes and P[5]A (Figure 4a). At higher anodic potentials, the efficiency of electropolymerization and stability of the coating decreased probably due to the formation of the products of monomer overoxidation. The proximity of the redox peaks did not allow for separating the signals of the macrocycle redox reactions that overlapped with those of the dyes.

The irreversible anodic peak recorded at 0.85–0.90 V corresponded to the formation of radical products of the dye oxidation initiating polymerization. If the upper potential was chosen below these values, no significant changes in voltammograms were found. With an increasing number of cycles, the peak current separation slightly increased due to the slower transfer of the monomers to the electrode. Moreover, another peak pair at −0.05–0.15 V appeared and grew with the number of cycles. It is commonly attributed to the formation of polymeric dyes that exerted similar redox behavior but at higher potentials against monomeric forms because of the steric factors [43].

When transferred to the phosphate buffer with no monomers, the electrode modified with polymeric dyes showed two pairs of broad peaks (Figure 4b). They were poorly resolved and probably corresponded to redox conversion of both polymeric dyes and P[5]A. In the absence of the macrocycle, the morphology of the peaks on voltammograms remained the same but the peak currents were about threefold lower. This might result from the involvement of all the components in the electron transfer chain and from the synergetic effect of the macrocycle and polyphenothiazines on the electron transfer. Vice versa, the signals of P[5]A alone (see a black line on Figure 4b corresponded to zero cycles of electropolymerization) were much lower and contained a pair of asymmetrical peaks with a bigger cathodic peak that corresponded to the reduction of benzoquinone units in the oxidized [5]A molecules. Domination of the reduction process can be referred to as the oxidation of the macrocycle with dissolved oxygen. Such behavior was earlier observed with the same coating (CB + P[5]A) in the assembly of DNA sensors [44]. In accordance with Figure 4b, most increases in the peak currents of the modified electrode have been reached at the 15th cycle and the maximal effect of electropolymerization was at −0.4 V.

### 3.2. Signal Measurement

The activity of the AChE is commonly monitored by the formation of thiocholine from the ATCh, a synthetic substrate added to the working solution. To characterize the effect of the electrode modification, the reaction was first considered with thiocholine obtained by mixing aqueous solutions of the enzyme and the substrate. The reaction was performed prior to the addition to the modified electrode. It was preliminarily established that 10 min. incubation was quite sufficient for full hydrolysis of the ATCh to thiocholine and acetic acid. Due to the instability of the thiocholine solution, the electrochemical measurements were performed with a freshly prepared solution containing 1 mM of thiocholine within an hour. To avoid the possible influence of the transducer components and uncertainty in the potential of pseudo-reference electrode screen-printed together with working and counter electrodes on plastic support, the measurements were made with a glassy carbon electrode modified following the protocol described above for the screen-printed electrode.

**Figure 5:**
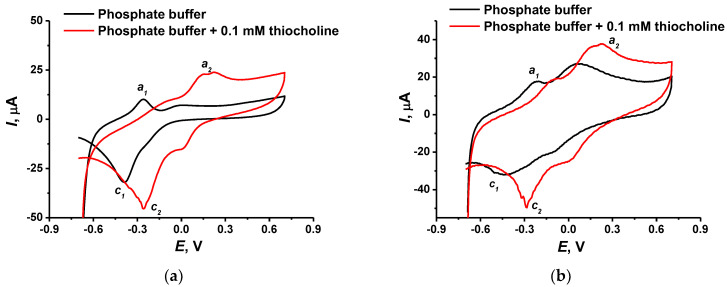
Cyclic voltammograms were recorded on a glassy carbon electrode covered with CB and adsorbed P[5]A (**a**) without and (**b**) with electropolymerized MB and thionine. Recorded in 0.01 M phosphate buffer + 0.1 M KCl containing 0.1 mM thiocholine. Measurements at pH = 7.0, 100 mV/s.

Figure 5 presents the comparison of cyclic voltammograms recorded on the modified glassy carbon electrode in phosphate buffer and that containing 0.1 mM thiocholine.

If the electrode was covered with CB and P[5]A, the addition of the thiocholine resulted in an increase in the peaks (*a*_1_, *c*_1_) attributed to the P[5]A and the appearance of a new peak (*a*_2_) related to the oxidation of thiocholine. The latter one corresponds to the 0.25 V and coincides with the conditions of the reactions reported earlier in similar research [23,24]. However, despite the significant difference in the peak currents, the voltammograms obtained in the presence of thiocholine were unstable and appropriate signals rapidly decreased in a series of consecutive measurements from the same solution. The electropolymerization of the MB and thionine decreased the response related to the thiocholine oxidation due to overlapping with the peaks of polymeric dyes. Meanwhile, the reduction peak current recorded at −0.27 V (*c*_2_) was quite stable and did not interfere with the currents related to other components of the surface layer. Thus, the following monitoring of the AChE activity was performed using the cathodic current corresponding to the P[5]A mediation of thiocholine redox conversion.

Other mediators of electron transfer utilized in the assembly of the electrochemical AChE biosensors amplify the anodic current related to thiocholine oxidation. The use of cathodic signals is the most significant advantage of the approach proposed. Most of the common interferences are oxidizable and hence can result in underestimation of the inhibition measured. Contrary to that, there are active species in the cathodic area and the signal is not disturbed by the by-products of the thiocholine oxidation able to passivate the electrode.

The immobilization of the AChE was performed using a well-known reliable method of carbodiimide binding. In them, terminal carboxylic groups of poly(lactic acid) were bonded to the amino groups of the protein. Although the number of accessible carboxylic groups on the inner walls of the reactor is limited, this cannot be a drawback for the inhibition determination. In such biosensors, the inhibitor concentration is mostly quantified using the inhibition degree. It is calculated as a ratio of inhibited enzyme to its total concentration in the reaction media. Assuming that the numerator of the fraction is constant, a decreased denominator means a high value of inhibition degree for the same quantity of an inhibitor. For this reason, the reproducibility of the signal and its stability during the measurement is of bigger importance than the absolute value of the current recorded.

**Figure 6:**
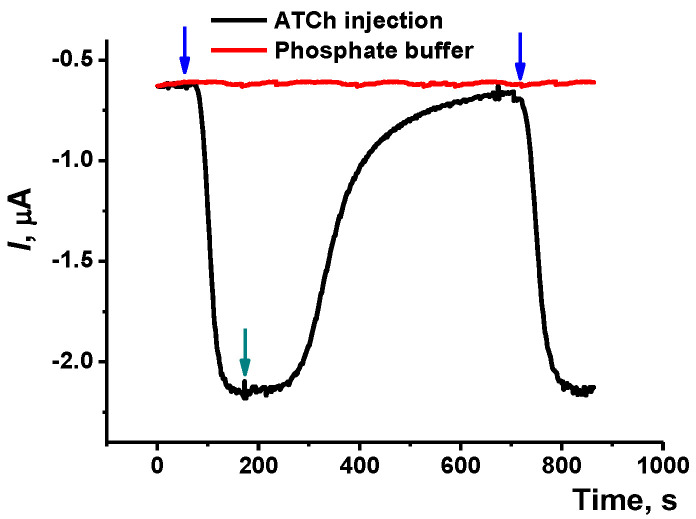
Dynamic response of the flow-through AChE biosensor to the 1.0 mM ATCh solution. Blue arrows correspond to the switching of the flow from the phosphate buffer to the substrate and the green arrow from the substrate to the phosphate buffer. Flow rate 0.2 mL/min.

The optimization of the AChE immobilization and measurement conditions was performed using a screen-printed electrode modified with CB + P[5]A and electropolymerized phenothiazine dyes. The conditions of modification corresponded to those described above. The electrode was fixed in a mounted flow-through cell and equalized with phosphate buffer solution. After that, the ATCh solution was pumped through the cell and the shift of the current was recorded in amperometric mode. The working potential corresponding to the maximum current in presence of ATCh with the pseudo-reference electrode was found to be −0.25 V (Figure S3). The dynamic response to the substrate is shown in Figure 6. As could be seen, switching flows resulted in rather fast changes in the current but the total response time is quite large—up to 15 min. This might result from a rather high dead volume of the cell. The distance between the inner wall of the reactor and the electrode surface is about 0.3 mm and the total volume of the cell is 36 μL. For the flow rate of 0.2 mL/min, the response time corresponds to the eightfold renewal of the solution in the cell. The attempts to decrease the height of the cell by additional membranes were unsuccessful because of the unpredictable positioning of a rather flexible screen-printed electrode against the enzyme location (replaceable reactor).

In continuous flow, the recorded current was stable within 6 h, the changes did not exceed 0.03 μA/h and were random without a pronounced trend. No tendencies of increasing response time were observed as well.

### 3.3. Optimization of the Measurement Conditions

**Figure 7:**
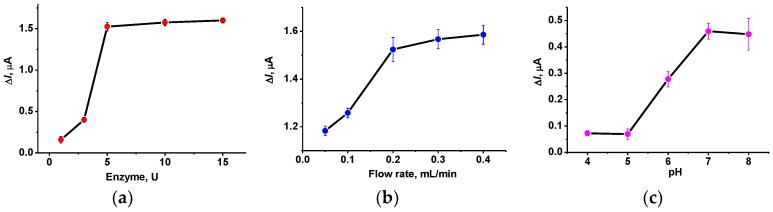
The dependence of the response of the AChE biosensor: (**a**) on the quantity of the enzyme taken for immobilization (ATCh 1.0 mM); (**b**) flow rate (AChE 5 U/biosensor, ATCh 1.0 mM); (**c**) pH (5 U of AChE, 0.2 mM ATCh). All the measurements at −0.25 V, average from three repetitions.

The main parameters of the AChE biosensor operation were optimized to find out the conditions corresponding to the maximum and most stable response. Appropriate dependencies are presented in Figure 7.

Full loading of the reactor with the AChE corresponded to 5 U per biosensor. The following increase in the enzyme quantities did not alter the response to 1.0 mM. It should be also noted that the repeatability of the signal was rather high and reached 1.1% for the same reactor and 2.5% for three individual reactors and the same modified screen-printed electrode. The increase in the flow rate of the substrate solution increased the signal up to 30–35% of its minimal value. This can be attributed to the faster transfer of the thiocholine to the electrode. The following measurement was performed at 0.2 mL/min. Regarding pH dependence of the response, the maximum (pH = 7–8) coincided well with the behavior of the native enzyme exerting maximal activity in basic media. However, the deviation of the signal was higher at pH 8.0 probably due to spontaneous hydrolysis of the ATCh, so the following inhibition measurements were performed at pH = 7.0.

Among other parameters, the concentration of the substrate is of main importance. It should be sufficient for full saturation of the enzyme active site so that any changes in the enzyme activity would affect the signal of the AChE biosensor. For this format of biosensor, the signal increased with the ATCh concentration to 1.0 mM (Figure S4). Thus, the following measurements were performed at this concentration of the substrate.

### 3.4. Reversible Inhibition Measurements

To assess the performance of the flow-through biosensor developed, two reversible inhibitors, donepezil [45] and berberine [46], were chosen. They are used as anti-dementia drugs and exert reversible inhibition on the AChE to compensate for the lack of acetylcholine observed in the acute stage of Alzheimer’s disease and other neurodegenerative diseases [45].

**Figure 8:**
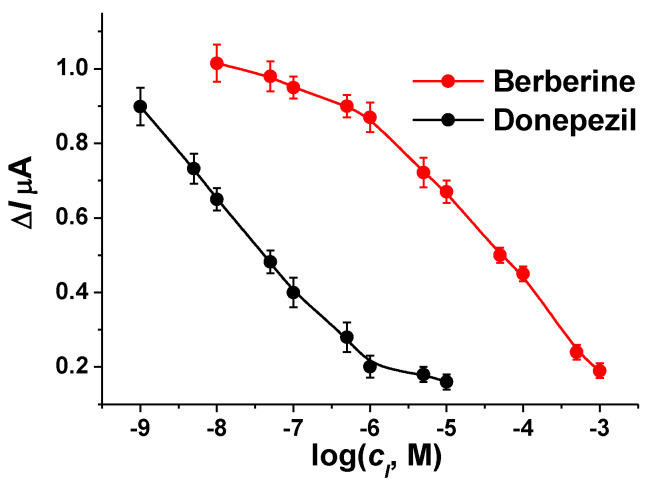
Calibration curves of donepezil and berberine were obtained with the flow-through AChE biosensor. AChE 5 U per biosensor, ATCh 1.0 mM, flow rate 0.2 mL/min, phosphate buffer, pH = 7.0. Average ± standard deviation for six measurements.

For both drugs, their solutions were mixed with 1.0 mM ATCh dissolved in phosphate buffer, pH = 7.0, and pumped through the cell. The stationary response has been reached at the third minute of pumping. The calibration curves are presented in Figure 8. As could be seen, increasing the concentration of the reversible inhibitor suppressed the signal down to zero in both cases. Meanwhile, the recovery of the AChE activity could be performed by 10 min washing the electrode with phosphate buffer even at high inhibitor concentrations. During the operation, up to 30 consecutive cycles of inhibition—recovery could be performed in continuous pumping of the solutions. The analytical characteristics of the drug determination are summarized in Table 1. The limit of detection (LOD) was calculated for a 6% decrease in the signal. This corresponded to the doubled deviation of the response measured. I_50_ corresponded to the inhibitor concentration resulting in a 50% decrease in the initial signal recorded in the absence of the inhibitor.

The results obtained are comparable with the characteristics of other AChE biosensors. The comparison is summarized in Table S1. Application of the second enzyme, choline oxidase, as well as the use of polyelectrolyte complexes with maximum mild immobilization of the AChE showed an advantage. Meanwhile, the only flow-through screening of reversible inhibition of the AChE with the working Au electrode covered with Au nanoparticles indicated a higher I_50_ value of donepezil determination [47].

The drugs tested are excreted from the human organism with urine. For this reason, we have prepared spiked samples of artificial urine (see Experimental). The interfering effect of the urine components was successfully eliminated by a 20-fold dilution. The comparison of the biosensor signal in phosphate buffer and diluted artificial urine is presented for berberine in Figure S5 as an example.

### 3.5. Irreversible Inhibition Measurements

**Figure 9:**
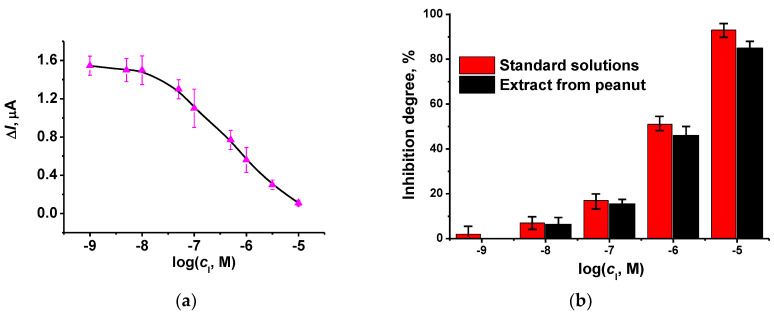
(**a**) Calibration curve of carbofuran obtained with the flow-through AChE biosensor. (**b**) Comparison of the inhibition degree recorded with standard solutions of carbofuran and diluted extracts from spiked peanut samples. Incubation 10 min, AChE 5 U per biosensor, ATCh 1.0 mM, flow rate 0.2 mL/min, phosphate buffer, pH = 7.0. Average ± standard deviation for six measurements.

Contrary to reversible inhibition, irreversible inhibition should be measured by consecutive addition of an inhibitor to the immobilized enzyme and only then of the substrate solution. This is due to the fact that the enzyme-substrate complex is much less sensitive or even insensitive to the irreversible inhibitor [48]. Such a requirement complicates the measurement protocol. We have tested carbofuran. Its solution was first pumped through the cell for 10 min. After that, the flow was switched to the 1.0 mM ATCh and the current was recorded as described above for reversible inhibition. In some experiments, the AChE reactivator, pralidoxime, was then pumped to restore the enzyme activity. In semi-logarithmic plots, the calibration curve is linearized in the range from 10 nM to 0.1 μM (LOD 5.0 nM) (Figure 9a). The appropriate calibration equation is presented below:

ΔI, μA = (−2.36 ± 0.11) − (0.49 ± 0.02) log(*C*_I_, M), *R*^2^ = 0.991, *n* = 7

The sensitivity of the biosensor signal toward carbofuran appeared to be lower than that of other AChE biosensors reported in the literature. The comparison of their analytical characteristics is presented in Table S2. Meanwhile, the only analog utilizing separate stages of enzyme inhibition and thiocholine detection [49] demonstrated a LOD of 20 nM and a general measurement time of more than 20 min.

There are several reasons explaining the lower sensitivity of carbofuran determination against the analogs. First, much longer incubation was used in the literature. In flow-through conditions, this was found not very effective because of possible deterioration of the surface layer. Not full removal of the substrate is a second reason. It was mentioned above that the design of the flow-through cell did not allow fast turnover of the solutions. Indeed, increased pumping of the buffer increased inhibition caused by carbofuran but not to an extent deserving prolongation of the measurement period. Indeed, the maximum acceptable concentration for carbofuran in drinking water is 90 µg/L (0.41 µM).

The flow-through AChE biosensor was tested on the spiked samples of peanuts. The preparation of the spiked samples and pesticide extraction were described in Experimental. Figure 9b shows the comparison of the inhibition detected for standard carbofuran solutions and diluted spied samples. The recovery was assessed as 91%. Thus, the biosensor developed can be applied for preliminary testing of carbofuran trace residues in peanuts.

Regarding the use of pralidoxime as a re-activator, treatment was found to be effective only for rather low inhibition of the AChE. If the decay of the current exceeded 50%, the repeated measurement of the inhibition resulted in the underestimated assessment of carbofuran content even though the response toward the substrate was about the same as prior to the contact of the biosensor with the inhibitor. It should also be noted that reactivation increases twofold the measurement time and could result in positive faults due to the own reversible inhibition effect of pralidoxime. For this reason, its application was estimated as undesirable.

### 3.6. Measurement Precision and Biosensor Lifetime

The drift of the signal of the flow-through biosensor to a repeated concentration of the substrate (1.0 mM ATCh) during six hours of the flow-through regime did not exceed 0.05 μA per measurement. This was similar to the background current drift (0.03 μA/h). Both values increased with the storage time of the reactor with the immobilized enzyme. The deviation grew regularly by about 10% per week. The general storage period of the reactor with immobilized enzyme was assessed as four months (dry conditions, 4 °C). Within a week, the reactor can also be stored at ambient temperature, however, the requirement of dry conditions remained critical. In addition, the repeatability of the response toward the substrate was calculated from the results of amperometric measurements performed using two sets of measurements, i.e., (1) six individual poly(lactic acid) reactors and the same screen-printed electrode modified with CB + P[5]A and polymeric MB and thionine, and (2) the same reactor and six modified screen-printed electrodes. Relative standard deviation was equal to 8 and 10%, respectively. The comparison of the results made it possible to conclude that mounting the parts of the flow-through cell was the main reason for the variation of the response.

Regarding inhibition measurements, relative decay of the signal was less sensitive to the storage period and replacement of the reactor/electrode. In all the cases, the deviation of inhibition degree was lower than 5%. It is a common estimate of the reproducibility of the signals of AChE biosensors.

Thus, the flow-through AChE biosensor developed showed quite satisfactory characteristics of inhibition measurements and storage stability acceptable for its application for fast and reliable inhibition detection.

## 4. Discussion

Separation of the steps of enzyme immobilization and signal detection have some advantages related to more flexible protocols of preparation and storage and the possibility of easy replacement of certain parts of the biosensor without the necessity of full changes in the surface layer. The use of the 3D printing technique and processible and cheap poly(lactic acid) allowed assembling a compact and simple flow-through biosensor where the advantages of P[5]A as electron transfer mediator and polymeric phenothiazine dyes as wiring compounds were demonstrated in the example of the determination of reversible and irreversible inhibitors. Consideration of the redox activity of electropolymerized matrices with implemented macrocycle made it possible to propose a new approach to signal measurement. Instead of anodic oxidation of thiocholine complicated with high overvoltage and electrode passivation, the reduction peak attributed to the P[5]A recovery was recorded as a measure of enzyme activity. In the presence of thiocholine released in enzymatic hydrolysis of ATCh, the current decreased due to the alternative path of chemical oxidation of the macrocycle. The mechanism of signal generation is confirmed by the fact that the dependence of the current on the rate of enzymatic reaction was observed only when the surface layer contained P[5]A molecules adsorbed on the CB particles.

The biosensor developed showed sufficient sensitivity toward donepezil and berberine, drugs used in the therapy of neurodegenerative diseases, and carbofuran as a representative of irreversible AChE inhibitors. Though the repeatability of signals and sensitivity toward inhibitors was slightly lower than those of “common” AChE biosensors, these drawbacks can be overcome by further improvement of the flow cell design. Even on this step, the analytical performance of the biosensor was quite sufficient for the determination of the inhibitors on the levels important for appropriate purposes. This was proved by the analysis of spiked samples of urine in the case of anti-dementia drugs and peanut extracts for carbofuran. The high efficiency of dilution for the elimination of interferences present in the samples is explained by several reasons. The high affinity of the drugs and pesticides to the AChE results from the design of their molecules to reach maximal binding to the enzyme active site. Natural inhibitors of AChE present in the samples exert a weak reversible effect, so that dilution shifts their concentrations to the values below the lower limits exerting inhibition on the enzyme. The use of the buffer also makes minimal pH changes and the influence of oligomeric substances is able to non-specifically bind the enzyme prior to its interaction with the analyte. The AChE biosensor showed a stable and reproducible signal both in model solutions and in spiked samples. Possible interference influence was eliminated by dilution of the samples. This offers good opportunities for the application of such flow-through biosensors as point-of-care (point-on-demand) devices outside of chemical laboratories.

## Figures and Tables

**Table 1:** Analytical characteristics of donepezil and berberine determination with the flow-through AChE biosensor.

Inhibitor	ΔI, μA = (*a* ± Δ*a*) − (*b* ± Δ*b*) log(*C*_I_, M)				Concentration Range, M	I_50_, nM	LOD, nM
	*a* ± Δ*a*	*b* ± Δ*b*	*n*	*R* ^2^			
Donepezil	−1.18 ± 0.04	0.23 ± 0.01	7	0.996	1 × 10^−9^–1 × 10^−6^	40	0.5
Berberine	−0.52 ± 0.03	0.24 ± 0.01	8	0.992	1 × 10^−6^–1 × 10^−3^	1240	120

## Data Availability

Not applicable.

## Supplementary Materials

References [47,49,50,51,52,53,54,55,56,57,58,59] are cited in the supplementary materials. The following supporting information can be downloaded at: https://www.mdpi.com/article/10.3390/bios12090676/s1, Figure S1: 3D design of the flow through cell for is manufacture by 3D printing; Figure S2: Photographs of the flow through cell and the screen printed electrode strip (a) disassembled and (b) assembled; Figure S3: Dependence of response of the flow-through AChE biosensor to the 1.0 mM ATCh solution on the applied potential of the working electrode; Figure S4: Concentration dependence of the signal of the flow-through AChE biosensor, 5 U AChE per biosensor, 0.2 mL/min, pH 7.0, electrode potential −0.25 V; Figure S5: Comparison of inhibition degree of berberine in phosphate buffer and 20-fold diluted artificial urine, flow-through AChE biosensor, 1.0 mM ATCh, 5 U AChE per biosensor, 0.2 mL/min, pH 7.0, electrode potential −0.25 V; Table S1: The comparison of the performance of the AChE biosensors for the determination of donepezil and berberine; Table S2: The comparison of the performance of the AChE biosensors for the determination of carbofuran.
