# Supplementary material for: Flow-Through Acetylcholinesterase Sensor with Replaceable Enzyme Reactor
Source: Biosensors (Basel). 2022 Aug 24;12(9):676. doi: 10.3390/bios12090676 (PMC9496324; doi:10.3390/bios12090676)
Supplement: Supplementary file 1 [file biosensors-12-00676-s001.zip › biosensors-1851788-supplementary.pdf]

Electronic Supporting Information

to the article of Alexey Ivanov, Dmitrii Stoikov, Ilziya Shafigullina, Dmitry Shurpik, Ivan Stoikov, and Gennady Evtugyn "Flow-Through Acetylcholinesterase Sensor with Replaceable Enzyme Reactor"

Modification of the screen-printed electrode

CB was first oxidized in concentrated nitric and sulfuric acid and then sonicated for 60 min. After that, it was centrifugated and washed with deionized water. After drying, oxidized CB was dispersed in propylene carbonate to final concertation of 0.66 mg/mL. Finally, 6.1 mg of pillar[5]arene were added to the dispersion and sonicated until the formation of a stable black colored solution. Then, 1 μL of the suspension was spread on the surface of working electrode and dried on air at 100°C for 30 min. Electropolymerization of the phenothiazine dyes was performed in a stationary electrochemical cell with electrochemical analyzer CHI 440B (CH Instruments Inc., Austin, TX, USA) in 0.01 M phosphate buffer containing 0.1 M KCl, pH = 7.0, by repeated cycling of the working potential in the range from -0.1 to 1.0. In optimal conditions, reaction media contained 0.1 mM thionine and 0.1 mM MB.

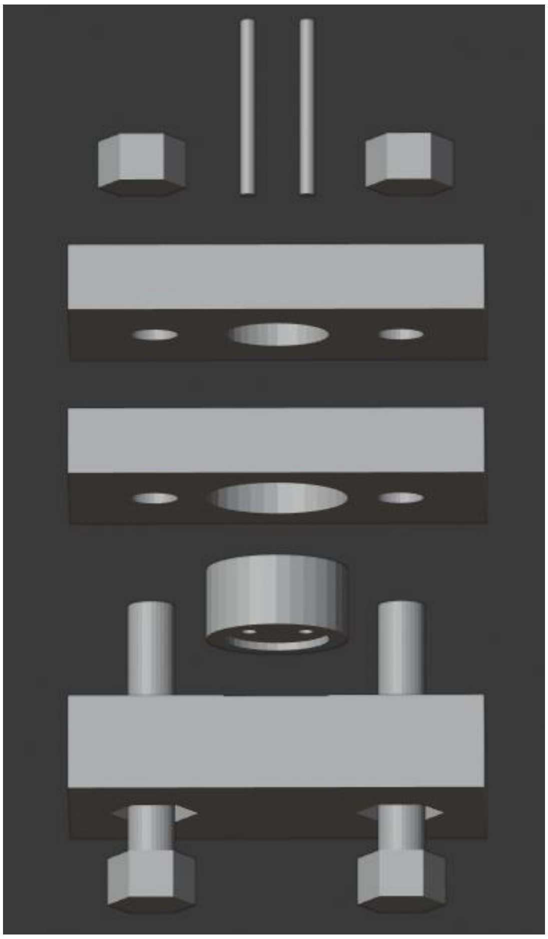

Figure S1. 3D design of the flow through cell for is manufacture by 3D printing

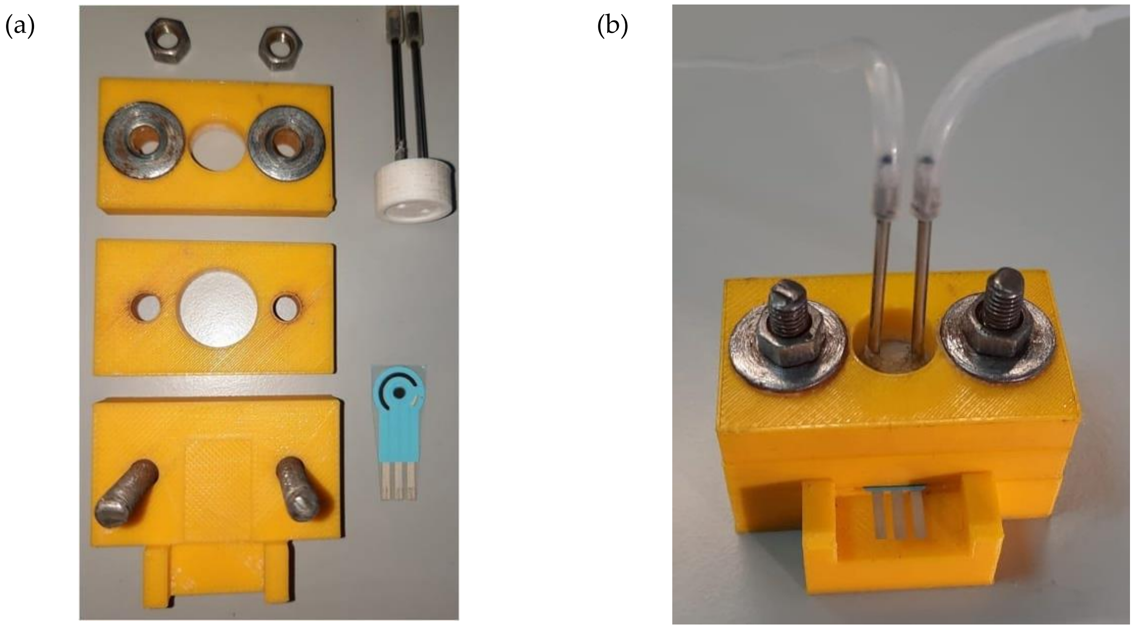

Figure S2. Photographs of the flow through cell and the screen printed electrode strip (a) disassembled and (b) assembled

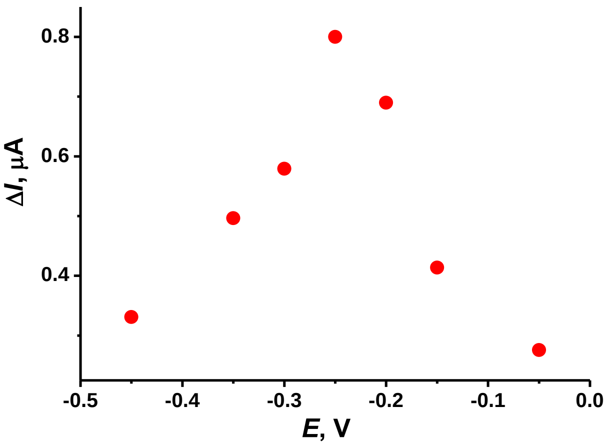

Figure S3. Dependence of response of the flow-through AChE biosensor to the 1.0 mM ATCh solution on the applied potential of the working electrode.

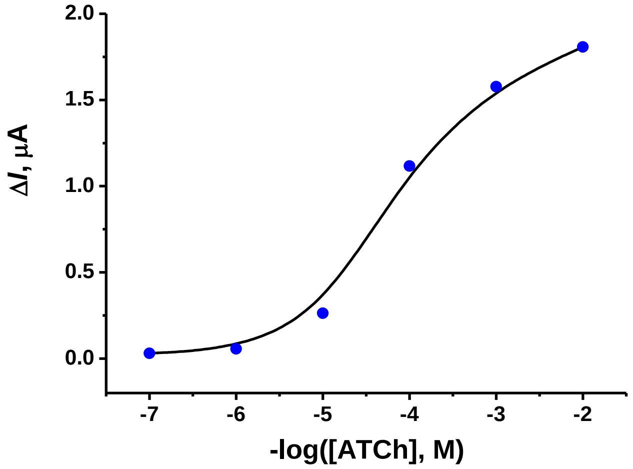

Figure S4. Concentration dependence of the signal of the flow-through AChE biosensor, 5 U AChE per biosensor, 0.2 mL/min, pH 7.0, electrode potential -0.25 V

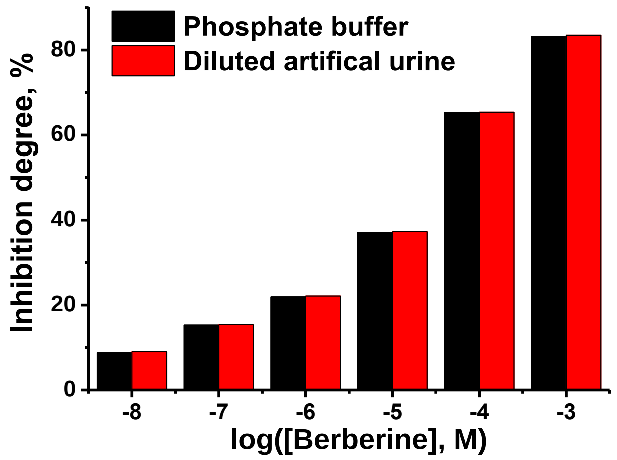

Figure S5. Comparison of inhibition degree of berberine in phosphate buffer and 20-fold diluted artificial urine, flow-through AChE biosensor, 1.0 mM ATCh, 5 U AChE per biosensor, 0.2 mL/min, pH 7.0, electrode potential -0.25 V

Table S1. The comparison of the performance of the AChE biosensors for the determination of donepezil and berberine.

Signal mode	Biosensor assembling	LOD, nM	I_{50}, nM	Ref.
		**Donezepil**		
Differential pulse voltammetry	Free AChE from human erythrocytes, Au screen-printed electrode	-	28	[S1]
Cyclic voltammetry	Covalent binding together with choline oxidase to electropolymerized thiophene derivative	0.07	-	[S2]
pH sensitive field effect transistor	Affine immobilization onto graphene via pyrene butanoic acid	-	47	[S3]
Amperometry in FIA mode	Covalent attachment to Au via cysteamine-glutaraldehyde binding	-	500	[S4]
Cyclic voltammetry	Implementation of the AChE in the polyelectrolyte complex onto glassy carbon electrode	0.46	24.7	[S5]
Amperometry in flow-through regime	Carbodiimide binding to poly(lactic acid) in the flow-through cell	0.5	40	This work
		**Berberine**		
Amperometry in FIA mode	Covalent attachment to Au via cysteamine-glutaraldehyde binding		6450	[S4]
Cyclic voltammetry	Implementation in polyelectrolyte complexes	70	590	[S5]
Amperometry in flow-through regime	Carbodiimide binding to poly(lactic acid) in the flow-through cell	120	1240	This work

[S1] Veloso, A,J.; Nagy, P.M.; Zhang, B.; Dhar, D.; Liang, A.; Ibrahim, T.; Mikhaylichenko, S.; Aubert, I.; Kerman, K. Miniaturized electrochemical system for cholinesterase inhibitor detection, *Anal. Chim. Acta*, **2013**, *774*, 73-78. DOI: 10.1016/j.aca.2013.02.033

[S2] Turan, J.; Kesik, M.; Soylemez, S.; Goker, S.; Kolb, M.; Bahadir, M.; Toppare, L. Development of an amperometric biosensor based on a novel conducting copolymer for detection of anti-dementia drugs. *J. Electroanal. Chem*. **2014**, *735*, 43-50. https://doi.org/10.1016/j.jelechem.2014.10.007.

[S3] Chae, M.-S.; Yoo, Y.K.; Kim, J.; Kim, T.G.; Hwang, K.S. Graphene-based enzyme-modified field-effect transistor biosensor for monitoring drug effects in Alzheimer's disease treatment. *Sens. Actuators B* **2018**, *272*, 448-458. https://doi.org/10.1016/j.snb.2018.06.010

[S4] Vandeput, M.; Parsajoo, C.; Vanheuverzwijn, J.; Patris, S.; Yardim, Y.; le Jeune, A.; Sarakbi, A.; Mertens, D.; Kauffmann, J.-M. Flow-through enzyme immobilized amperometric detector for the rapid screening of acetylcholinesterase inhibitors by flow injection analysis. *J. Pharm. Biomed. Anal.* **2015**, *102*, 267-275. DOI: 10.1016/j.jpba.2014.09.012

[S5] Ivanov, A.; Davletshina, R.; Sharafieva, I.; Evtugyn, G. Electrochemical biosensor based on polyelectrolyte complexes for the determination of reversible inhibitors of acetylcholinesterase. *Talanta* **2019**, *194*, 723-730. https://doi.org/10.1016/j.talanta.2018.10.100

Table S2. The comparison of the performance of the AChE biosensors for the determination of carbofuran.

Signal mode	Biosensor assembling	LOD, nM	Ref.
Square-wave voltammetry	Screen-printed electrode, the inhibitor solution is pumped through the tube with magnetic beads modified with the AChE, then, thiocholine was separated and measured	20	[S6]
Cyclic voltammetry	Covalent binding to CB-chitosan dispersion, measurements in the presence of iodide ions as mediators	0.6	[S7]
Cyclic voltammetry	Affine immobilization via concanavalin A, electrode modified with polydopamine, reduced graphene oxide and Au nanoparticles	50	[S8]
Cyclic voltammetry	AChE immobilized with bovine serum albumin on ferrite particles	3.6	[S9]
Square-wave voltammetry	AChE immobilized on carbon nanospheres	0.3	[S10]
Differential pulse voltammetry	AChE immobilized in nafion – chitosam matrix, Prussian Blue as mediator	2.5	[S11]
Differential pulse voltammetry	Enzyme implemented in 3D graphene oxide network / multiwalled carbon nanotubes	0.07	[S12]
Amperometry in flow-through regime	Carbodiimide binding to poly(lactic acid) in the flow-through cell	5.0	This work

[S6] Kostelnik, A.; Kopel, P.; Cegan, A.; Pohanka, M. Construction of an acetylcholinesterase sensor based on synthesized paramagnetic nanoparticles, a simple tool for neurotoxic compounds assay. *Sensors* **2017**, *17*, 676. https://doi.org/10.3390/s17040676.

[S7] Soulis, D.; Trigazi, M.; Tsekenis, G.; Chandrinou, C.; Klinakis, A.; Zergioti, I. Facile and low-cost SPE modification towards ultra-sensitive organophosphorus and carbamate pesticide detection in olive oil. *Molecules* **2020**, *25*, 4988. https://doi.org/10.3390/molecules25214988.

[S8] Li, Y.; Li, Y.; Yu, X.; Sun, Y. Electrochemical determination of carbofuran in tomatoes by a concanavalin A (Con A) polydopamine (PDA)-reduced graphene oxide (rGO)-gold nanoparticle (GNP) glassy carbon electrode (GCE) with immobilized acetylcholinesterase (AChE). *Anal. Lett.* **2019**, *52*, 2283-2299. https://doi.org/10.1080/00032719.2019.1609490

[S9] Jeyapragasam, T.; Saraswathi, R. Electrochemical biosensing of carbofuran based on acetylcholinesterase immobilized onto iron oxide–chitosan nanocomposite. *Sens. Actuators B* **2014**, *191*, 681-687. https://doi.org/10.1016/j.snb.2013.10.054.

[S10] Cai, J.-R. ; Zhou, L.-N.; Han, E. A sensitive amperometric acetylcholine biosensor based on carbon nanosphere and acetylcholinesterase modified electrode for detection of pesticide residues. *Anal. Sci.* **2014**, *30*, 669-673. https://doi.org/10.2116/analsci.30.669

[S11] Zhai, C.; Sun, X.; Zhao, W.; Gong, Z.; Wang, X. Acetylcholinesterase biosensor based on chitosan/prussian blue/multiwall carbon nanotubes/hollow gold nanospheres nanocomposite film by one-step electrodeposition. *Biosens. Bioelectron*. **2013**, *42*, 124-130. https://doi.org/10.1016/j.bios.2012.10.058

[S12] Li, Y.; Zhao, R.; Shi, L.; Han, G.; Xiao, Y. Acetylcholinesterase biosensor based on electrochemically inducing 3D graphene oxide network/multi-walled carbon nanotube composites for detection of pesticides. *RSC Adv*. **2017**, *7*, 53570-53577. https://doi.org/10.1039/C7RA08226F.
